# Regulation of a LATS-homolog by Ras GTPases is important for the control of cell division

**DOI:** 10.1186/1471-2121-15-25

**Published:** 2014-07-01

**Authors:** Annette Müller-Taubenberger, Peter M Kastner, Michael Schleicher, Parvin Bolourani, Gerald Weeks

**Affiliations:** 1 Anatomy III - Cell Biology, Ludwig Maximilian University of Munich, Schillerstr. 42, 80336 Munich, Germany; 2 Department of Microbiology and Immunology, University of British Columbia, Vancouver, BC V6T 1Z3, Canada

**Keywords:** Cell division, *Dictyostelium discoideum*, Ras GTPase, LATS kinase

## Abstract

**Background:**

Nuclear Dbf-related/large tumor suppressor (NDR/LATS) kinases have been shown recently to control pathways that regulate mitotic exit, cytokinesis, cell growth, morphological changes and apoptosis. LATS kinases are core components of the Hippo signaling cascade and important tumor suppressors controlling cell proliferation and organ size in flies and mammals, and homologs are also present in yeast and *Dictyostelium discoideum*. Ras proto-oncogens regulate many biological functions, including differentiation, proliferation and apoptosis. Dysfunctions of LATS kinases or Ras GTPases have been implicated in the development of a variety of cancers in humans.

**Results:**

In this study we used the model organism *Dictyostelium discoideum* to analyze the functions of NdrC, a homolog of the mammalian LATS2 protein, and present a novel regulatory mechanism for this kinase. Deletion of the *ndrC* gene caused impaired cell division and loss of centrosome integrity. A yeast two-hybrid analysis, using activated Ras proteins as bait, revealed NdrC as an interactor and identified its Ras-binding domain. Further *in vitro* pull-down assays showed that NdrC binds RasG and RasB, and to a lesser extent RasC and Rap1. In cells lacking NdrC, the levels of activated RasB and RasG are up-regulated, suggesting a functional connection between RasB, RasG, and NdrC.

**Conclusions:**

*Dictyostelium discoideum* NdrC is a LATS2-homologous kinase that is important for the regulation of cell division. NdrC contains a Ras-binding domain and interacts preferentially with RasB and RasG. Changed levels of both, RasB or RasG, have been shown previously to interfere with cell division. Since a defect in cell division is exhibited by NdrC-null cells, RasG-null cells, and cells overexpressing activated RasB, we propose a model for the regulation of cytokinesis by NdrC that involves the antagonistic control by RasB and RasG.

## Background

Nuclear Dbf-related/large tumor suppressor (NDR/LATS) kinases have been shown recently to control pathways regulating mitotic exit, cytokinesis, cell proliferation, morphological changes and apoptosis [1-3]. Work over the past decade has revealed that LATS kinases are core components of the Hippo signaling pathway that has been shown to have a tumor suppressor function and to control tissue growth in flies and mammals [4-8]. Loss of NDR/LATS kinase activity has been related to the development of various human malignancies [1,4,9].

The Hippo pathway was identified first by genetic screens in *Drosophila melanogaster*. The Ste20 kinase Hippo (MST1/2 in mammals) and the NDR family kinase Warts (LATS1/2 in mammals) constitute the core of the signaling cascade. The elucidation of the mechanisms that regulate the Hippo pathway and the identification of interactors contributes to our understanding how growth and organ size in flies and mammals are controlled and why misregulation leads to the formation of cancer. Recent studies in *Drosophila* and mammalian cells have suggested that LATS kinases are involved in the density-dependent control of cell proliferation through a cell morphology-based mechanism which is mediated by stress fibers and cooperates with a cell adhesion-based mechanism [10-12].

Homologs of the Hippo pathway components have been shown to be present in yeast [5,13], *Dictyostelium discoideum*[14], and *Capsaspora owczarzaki*[15], thus providing an opportunity for the genetic analysis of the essential and evolutionary functions of these important regulators. In particular *Dictyostelium* is an easily accessible eukaryotic model system to gain insights into a variety of basic cellular processes, including the regulatory machinery controlling cell division [16,17]. The LATS/NDR family of *Dictyostelium* consists of two LATS-related kinases, NdrC and NdrD, as well as two NDR-related kinases, NdrA and NdrB [18,19]. In the present study, we have explored the function of NdrC, and provide evidence that NdrC plays an important role in cell division. Based on the data presented, we propose that its activity is antagonistically controlled by RasB and RasG, two members of the Ras subfamily of GTPases.

## Results and discussion

### Identification of NdrC as a Ras GTPase interacting protein

**Figure 1:**
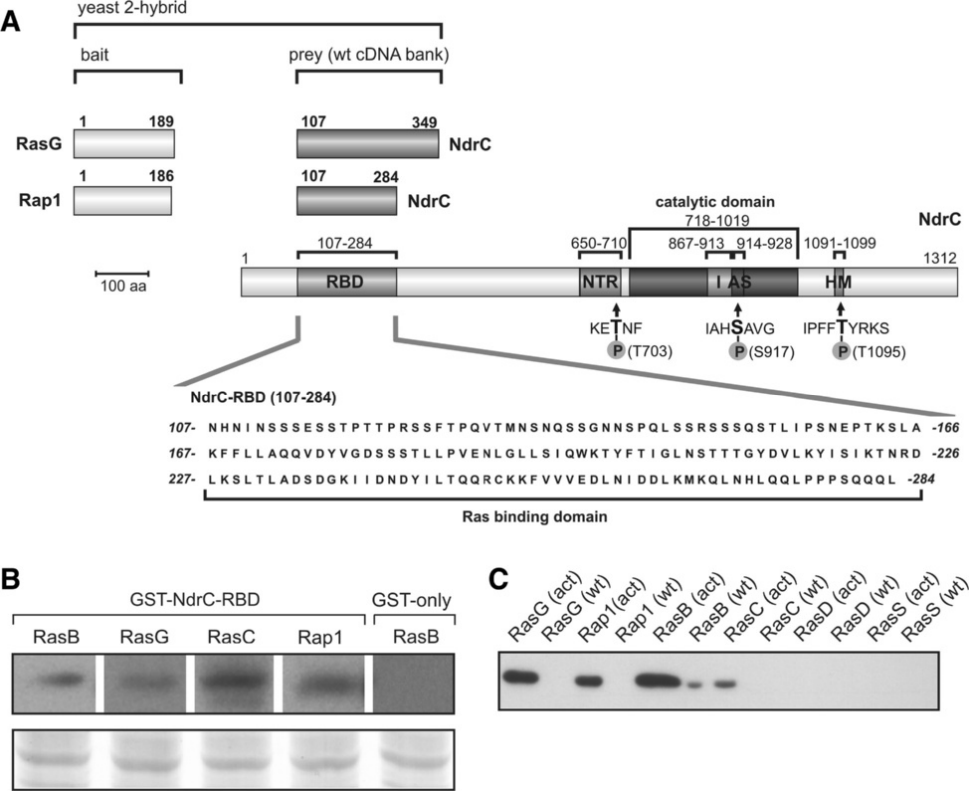
**NdrC interacts with Ras proteins. A**. In yeast two-hybrid experiments with various activated Ras proteins as bait, NdrC was revealed as a strong interactor of RasG and Rap1. Domain organization of NdrC, and mapping of the Ras binding domain (RBD, aa 107–284), NTR (N-terminal regulatory domain, aa 650–710), phosphorylation site (T703), catalytic domain (kinase domain aa 718–1019, subdomains I-X), I (insert, aa 867–913), AS (activation segment, aa 914–928; regulatory phosphorylation site S917), HM (hydrophobic motif aa 1092–1099; phosphorylation site T1095). **B**. Binding of GST-NdrC-RBD to Ras proteins *in vivo*. The RBD of NdrC was used to detect Ras proteins in *Dictyostelium* cell lysates as described in *Methods*. The bound material was analyzed by Western blotting using antibodies specific to RasB, RasG, RasC and Rap1 (upper panel). GST-only was tested in all pull-down experiments, and consistently there was no binding (here shown only for RasB). Equal sample loading was verified by staining of a duplicate gel with Coomassie Blue (lower panel); only the range of the strongest band is shown. **C**. Representative Western blot showing the pull-down of His-tagged Ras proteins by GST-tagged NdrC-RBD. Recombinant His-tagged Ras proteins (constitutively GTP- or GDP-bound) were allowed to bind *in vitro* to GST-tagged bacterially expressed NdrC-RBD. For details of the quantitative assay please see *Methods*. The amount of bound Ras proteins was detected by Western blotting using an anti-His Tag antibody.

*Dictyostelium discoideum* NdrC (DDB0349842) belongs to the LATS/NDR kinase family, which constitutes a subgroup of AGC (protein kinase A/G/C-related) kinases [18,20]. The *Dictyostelium* LATS/NDR family consists of four kinases, two ‘shorter’ NDR kinases (NdrA/B), and two ‘larger’ LATS/NDR-related kinases (NdrC/D) that are characterized by an extended N-terminus [19]. Similarly, the mammalian LATS/NDR kinase family is subdivided into two ‘larger’ LATS kinases (LATS1/2) and two ‘shorter’ NDR kinases (NDR1/2) (Additional file 1: Figure S1A). The *Dictyostelium* NdrC kinase is made up of 1,312 amino acids (147 kDa), and its protein sequence comprises the general features described for other LATS/NDR kinases, which include an N-terminal regulatory domain (NTR), an insert sequence (I) between subdomain VII and VIII of the catalytic domain, an activation segment (AS) as well as a conserved hydrophobic motif (HM) (Figure 1A, Additional file 1: Figure S1B).

When the *Dictyostelium* GTPases RasG, RasC and Rap1 were employed as bait in a yeast two-hybrid screen, NdrC was identified as a novel interacting protein that exhibited strong positive interactions with all three proteins. This screen also revealed that RasG and Rap1 exhibited strong positive interactions with the previously described Ras-binding domain (RBD) containing proteins PL3K and Rip3. In contrast, NdrC was the only protein that bound RasC in the yeast two-hybrid screen. The RBD of NdrC was localized between amino acid residues 107 and 284, the sequence that interacted with all three Ras proteins in the yeast two-hybrid assays (Figure 1A and data not shown). The minimal Ras-binding domain of NdrC was not defined further by additional experiments. A bioinformatics analysis did not reveal evidence of a RBD sequence in this region, but this is not surprising since the RBD sequences of a number of other RBD proteins were not detected by bioinformatic analyses [21].

Ras protein binding to NdrC was confirmed by pull-down assays using the identified RBD of NdrC tagged to GST and *Dictyostelium* lysates (Figure 1B). Bound Ras proteins were detected by Western blot analysis using specific anti-Ras polyclonal antibodies. The experiment revealed that the NdrC-RBD interacted not only with RasG, RasC and Rap1, but also with RasB (Figure 1B). This result was confirmed and extended by pull-down experiments using GST-tagged NdrC-RBD in combination with His-tagged Ras subfamily proteins that allowed a more quantifiable comparison of the binding. These interaction studies showed that NdrC bound to the activated Ras proteins but not to the wild type (Figure 1C), although the wild-type proteins did bind in the presence of GTPγS (data not shown). NdrC bound best to activated RasG and RasB, with lower levels of binding to activated Rap1 and RasC (Figure 1C). NdrC did not interact with either RasD or RasS (Figure 1C).

### *ndrC*-null cells exhibit a severe defect in cytokinesis and aberrant numbers of centrosomes

**Figure 2:**
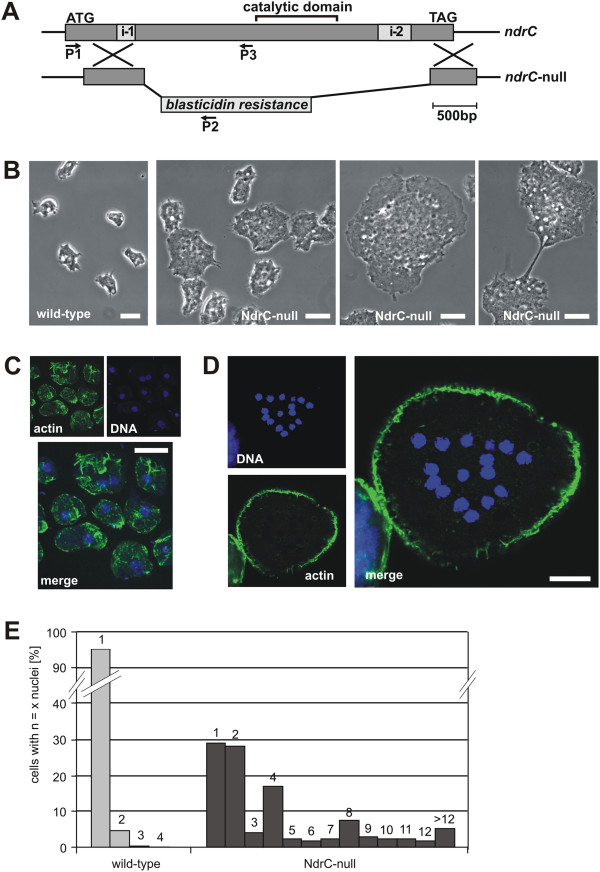
**NdrC-null cells are impaired in cell division. A**. Gene replacement of *ndrC* (DDB0219984) by a blasticidin-S resistance cassette. P1-P2 and P1-P3 indicate the primer combinations that were used initially to identify gene knockouts by PCR. **B**. Live-cell microscopy of wild-type (left) and *ndrC*-null cells. *ndrC*-null cells are much larger than wild-type cells, and often divide by traction-mediated cytofission (as shown in the right image). **C**. Fixed wild-type cells stained with TRITC-phalloidin for actin and TO-PRO-3 for DNA. **D**. Fixed *ndrC*-null cell stained with TRITC-phalloidin for actin and TO-PRO-3 for DNA. **E**. Histogram showing the percentage of cells carrying the indicated numbers of nuclei in wild-type and *ndrC*-null mutants. Cells were grown in Petri dishes, fixed, stained, and for each strain the nuclei of >500 cells were counted. The counting was repeated in an independent experiment with almost identical results. Bars, 10 μm.

To investigate the cellular function of NdrC, a knockout strain of the *ndrC* gene was generated by homologous recombination using a disruption construct carrying an inverted blasticidin-S resistance cassette within the partial *ndrC* gene sequence (Figure 2A). The most striking phenotype of the *ndrC*-null cells when compared to the wild type was that the majority of mutant cells were larger and multinucleated (Figure 2B). When wild-type and *ndrC*-null cells were fixed and stained with TO-PRO-3 to visualize the nuclei, and counterstained for actin using Alexa Fluor 488-coupled phalloidin, the increased number of nuclei in the mutant was clearly revealed (Figure 2C, D), and this increase was quantified by nuclei counts of fixed, DAPI-stained cell preparations (Figure 2E). The percentage of mono-nucleated cells in wild type was 95% compared to only 29% in *ndrC-*null cells (Figure 2E). The number of nuclei per cell in the *ndrC-*nulls peaked at 1, 2, 4 and 8 indicating concerted mitosis (Figure 2E).

An analysis of living cells lacking NdrC revealed that they were able to perform mitosis, but an impairment of cell division resulted in the formation of multinuclear cells. Frequently, putative daughter cells were still connected by thin cellular bridges suggesting division by cytofission rather than mitosis-coupled cytokinesis (Figure 2B, right). The generation times, measured for *ndrC*-null and wild-type cells were almost identical, regardless of whether cells were grown in shaking culture in rich axenic medium or on a solid substrate with bacterial lawns (Additional file 2: Figure S2). Since *ndrC-*null cells are considerably larger than wild-type cells, their cell masses increase at considerably faster rates indicating an additional defect in the control of cell growth.

**Figure 3:**
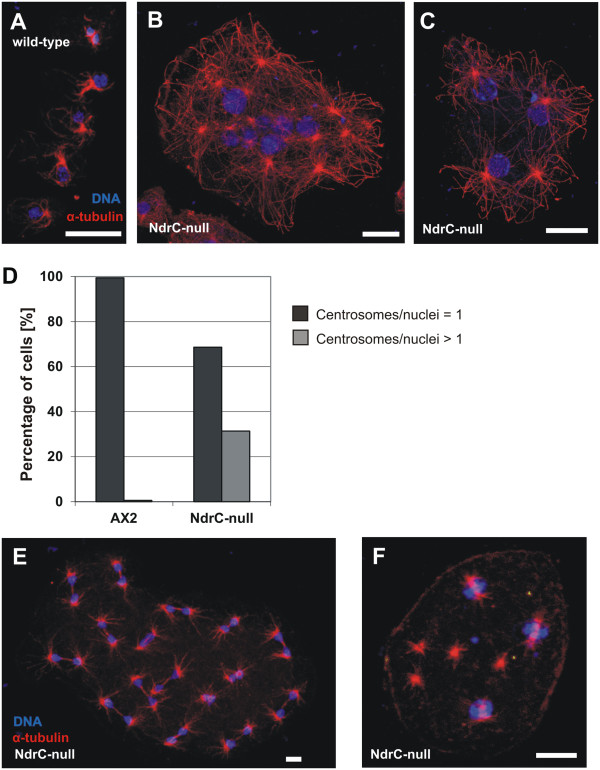
**Cells lacking NdrC show centrosomal aberrations. A**. In comparison to wild-type, **B**, **C **multinucleated *ndrC*-null cells frequently show supernumerous centrosomes. **D**. Histogram depicting the percentage of normal (centrosomes/nuclei = 1) and aberrant (centrosomes/nuclei > 1) centrosomes in wild-type and *ndrC*-null cells. **E**, **F**. Visualization of mitotic spindles in fixed *ndrC*-null cells by immunolabeling with anti-α-tubulin antibodies, and staining of DNA with TO-PRO-3. Spindle formation occurs synchronously in multinucleate cells **(E)**, as well as in multinucleate cells with supernumerous centrosomes **(F)**. Bars 5 μm in **(A)**, and, 10 μm in **(B)**, **(C)**, **(E)**, and **(F)**.

Inspection of multinucleated *ndrC*-null cells, fixed and stained with TO-PRO-3 for visualization of nuclei and immunostained with anti-α-tubulin antibodies to visualize microtubules and centrosomes, revealed a high percentage of mutant cells carrying increased numbers of centrosomes (Figure 3). Frequently, the supernumerous centrosomes were not connected to the nuclei (Figure 3B). To quantify the disruption of centrosome integrity, the ratio of centrosomes visualized by anti-α-tubulin staining to nuclei visualized by DAPI staining, was determined for wild-type and *ndrC*-null cells (Figure 3D). About one third (31%) of the *ndrC-*null cells had more than one centrosome per nucleus, compared to only 0.6% in wild-type cells. *ndrC*-null cells with evenly distributed centrosomes underwent mitosis in a synchronized manner (concerted mitosis) (Figure 3E), but although the increased ratio of centrosomes per nuclei did not affect the ability of cells to perform concerted mitosis, the unattached surplus centrosomes were not capable of forming mitotic spindles (Figure 3F).

Aberrant numbers of centrosomes have also been observed for knockout strains of mouse LATS2 [22,23] and human NDR1/2 [3,24]. The cell division defect in mammalian cells has been attributed to a disruption of the signaling cascade controlling cytokinesis on the one hand [25], and a control of nuclear division on the other [26]. Human LATS1 was also reported to act as mitotic exit network kinase [27], responsible for regulation of the G2/M-arrest [28]. In addition, the *Drosophila* LATS-homolog Warts regulates mitotic progression [29]. However, despite these clear indications for a role of NDR/LATS kinases in regulation of the cell cycle and/or cell division, the underlying mechanisms are largely unknown.

### Subcellular localization of NdrC

**Figure 4:**
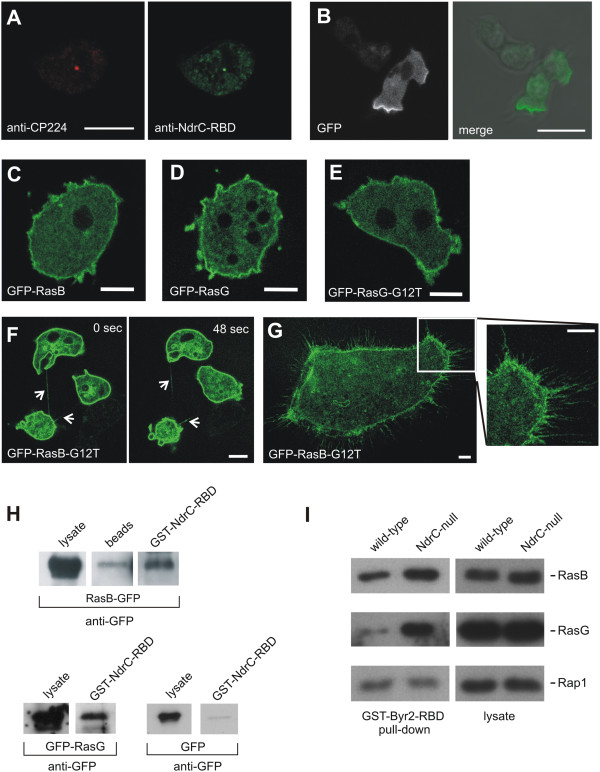
**Localization and interaction of NdrC with Ras proteins. A**. NdrC localizes to the centrosome. Fixed *Dictyostelium* cells were immunolabeled with centrosome-specific monoclonal anti-CP224 antibodies, and polyclonal anti-NdrC antibodies. Primary antibodies were detected with anti-mouse Alexa Fluor-568 and anti-rabbit Alexa Fluor-488 antibodies. In NdrC-null cells, no staining with anti-NdrC antibodies was detected. Bar, 10 μm. **B**. Live-cell imaging of a *Dictyostelium* cell expressing GFP-NdrC-RBD. Left image, GFP-NdrC-RBD signal; right image, merge. Bar, 10 μm. **C**. Live-cell imaging of GFP-RasB expressing wild-type cells shows localization of GFP-RasB to the cell cortex. **D**. GFP-RasG localization to the cortex of wild-type cells, shown by live cell microscopy. **E**. GFP-RasG(G12T) localizes to the cortex and filopodia of wild-type cells. **F**. Cytofission of GFP-RasB(G12T) expressing wild-type cell. **G**. GFP-RasB(G12T) expressed in wild-type cells localizes to the cortex and filopodia (image enlargement on the right), and results in enlarged multinucleate cells. Bars in **(C)**-**(G)**, 5 μm. **H**. GST-NdrC-RBD pull-down of RasB tagged to GFP shows interaction and thereby activity of the GFP-tagged Ras-GTPase. GFP-tagged RasG overexpressed in wild-type cells interacts with the GST-NdrC-RBD. The levels of bound RasG were detected by Western blotting using anti-GFP antibodies. **I**. Levels of activated Ras proteins in wild-type compared to *ndrC*-null cells. Total cell extracts from wild-type or *ndrC*-null cells were bound to GST-Byr2-RBD as described in *Methods*. The amount of activated Ras proteins pulled down or of total Ras protein in the lysate was determined by Western blotting using specific polyclonal antibodies against RasB, RasG, and Rap1. The data shown is for a single experiment, but similar results were obtained in two other experiments.

Gene expression profiling data indicated that NdrC is expressed at very low levels throughout the developmental cycle of *Dictyostelium* (http://dictyexpress.biolab.si) [30]. In order to specify the cellular localization of NdrC, polyclonal antibodies were raised against NdrC. Immunolabeling experiments with fixed *Dictyostelium* cells indicated that NdrC is preferentially enriched at the centrosome (Figure 4A). This localization was confirmed by co-staining with a monoclonal antibody directed against a genuine centrosomal component, the corona protein CP224 [31]. The centrosomal localization of NdrC was detectable only in a subfraction of cells, suggesting a cell cycle-dependent enrichment. Expression of full-length NdrC with a N-terminal GFP-tag confirmed the localization at the centrosome. NdrC had not been detected in a previous proteome screen of the *Dictyostelium* centrosome [32], but was found to co-purify with centrosomes that were isolated from *Dictyostelium* wild-type and cells expressing GFP-NdrC (Additional file 3: Figure S3).

Over-expression of a fusion protein comprising GFP and the N-terminal 300 amino acids of NdrC containing the RBD domain, showed a very similar localization as that reported previously for the Ras-binding domain of human Raf1 that relocates upon stimulation to active zones of the cell cortex [33,34] (Figure 4B). This finding indicates a preferential binding of RBD of NdrC to membrane-associated Ras proteins. A GFP-fusion protein comprising the catalytic domain and the C-terminus of NdrC (amino acid residues 435 to 1312) localized to the cytoplasm and showed no specific enrichment (Additional file 4: Figure S4).

### Putative control of NdrC by Ras GTPase family members

We have shown previously that *rasG-*null cells or cells overexpressing RasB-(G12T) exhibit a multinucleated cell phenotype that is similar to the one exhibited by the *ndrC-*null cells [35,36]. In addition, severe defects in cytokinesis in cells overexpressing the exchange factor RasGEF-Q, which acts specifically upstream of RasB have been described [37]. Given that NdrC contains a RBD, it is conceivable that RasB or RasG exert regulatory functions on NdrC.

To further investigate the possible roles of RasG or RasB in NdrC function, we determined the subcellular localization of RasG and RasB. Wild-type cells overexpressing GFP-tagged RasB or RasG were normal in respect to cell size and nuclei number, and the GFP-tagged proteins predominately localized to the cortex (Figure 4C, D). The GFP-tagged activated forms of the proteins, RasB-(G12T) and RasG-(G12T), also localized predominately to the cortex (Figure 4E-G). However, whereas RasG-(G12T) overexpression did not affect the cell phenotype (Figure 4E), overexpression of RasB-(G12T) caused disturbed cytokinesis characterized by an impairment of cells to sever the connection between daughter cells (Figure 4F). This resulted in enlarged, multinucleated cells (Figure 4F), a defect similar to that described previously for cells expressing an untagged RasB-(G12T) [35].

Both, GFP-tagged RasB and GFP-RasG bound to the GST-NdrC-RBD, as shown by *in vitro* pull-down assays of wild-type AX2 cells expressing GFP-tagged Ras proteins (Figure 4H). Although NdrC also binds RasC and Rap1, cell division is not affected by either the loss or up-regulation of Rap1 [38], or by the loss or up-regulation of RasC [39]. Thus the significance of the binding of Rap1 and RasC to NdrC remains to be established.

Further evidence for a possible functional connection between the Ras-GTPases and NdrC was obtained by measuring the levels of activated Ras protein in the *ndrC-*null cells by Western blot analysis of GST-Byr2-RBD pull-downs using specific antibodies for the Ras family members. Levels of activated RasB and RasG were increased in the absence of NdrC compared to wild-type cells, indicating that NdrC acts as a negative regulator for activation of RasG and RasB (Figure 4I). In contrast, the levels of activated Rap1 and the total levels of all the Ras GTPases were found to be identical in lysates of wild-type and NdrC*-*null cells (Figure 4I). The mechanism involved in the feedback inhibition of RasG and RasB activation by NdrC, presumably at the level of RasGEF activity, is not known at this time, but it appears to be a general mechanism for downstream Ras effectors in *Dictyostelium*, since similar results have been observed for *pik* null and *ripA* null cells.

**Figure 5:**
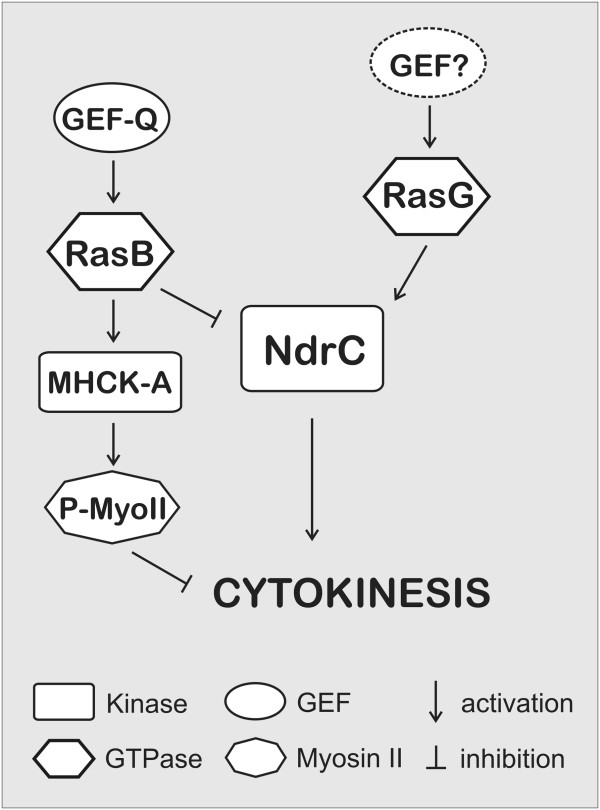
Model suggesting the regulation of NdrC by Ras GTPases as explained in the text.

The findings that an absence of RasG and the overexpression of activated RasB both result in a multinucleate phenotype suggest the possibility that RasB and RasG play antagonistic roles in regulating NdrC during cytokinesis (Figure 5). The multinucleate phenotype of *rasG*-null cells [36] is consistent with a role for RasG in activating NdrC, whereas the multinucleate phenotype generated by over-expression of either activated RasB [35], or the RasB-specific exchange factor GefQ [37], indicates that RasB is a negative regulator of cytokinesis and cell division.

The regulation of NdrC by RasG and RasB implies that these Ras proteins would also be localized at the centrosome or in the nucleus. When cells were labeled with GFP-tagged Ras proteins, we find wild-type RasB and RasG, as well as constitutively active forms of the two proteins, are localized predominantly at the plasma membrane. This result contrasts somewhat with a previous study, using antibody staining, that revealed RasB clearly localized at the nucleus [35]. When we stained cells with an antibody highly specific to RasG, the majority of the stain was associated with the cell cortex, but some stain was also detected in the cytoplasm and in the nucleus (Additional file 5: Figure S5). These results imply that only a small proportion of the cellular RasG and RasB is localized in the nucleus, and are consistent with RasG and RasB both having other functions in the cell. In fact, RasG is not only involved in cytokinesis, but also in folate chemotaxis, in maintenance of cell shape, and in cell motility [40]. In addition, it has been shown recently that RasG, acting through PI3K, is important for pinocytosis [41]. Evidence for other specific functions for RasB is less direct, but *gefQ-*null cells exhibit myosin-II over-assembly and defects in polarity [37], implying a possible role for RasB in these functions, and *rasB* appears to be an essential gene [35], suggesting an additional vital function for RasB.

## Conclusions

We present evidence for a novel mechanism for the regulation of cell division in *Dictyostelium,* involving the LATS2-homolog NdrC and members of the Ras subfamily of GTPases, RasB and RasG. We propose an antagonistic regulation, whereby the NdrC is activated by RasG and inactivated by RasB. The previously described interaction between the *Schizosaccharomyces pombe* Cdc42, a small GTPase, and Orb6, a member of the LATS/NDR group of kinases [42] suggests the possible generality of this type of mechanism, and it will be important to investigate whether the mammalian LATS kinases are also regulated by small GTPases during cell division.

## Methods

### Cell culture, gene replacement and transformation of *Dictyostelium*

Cells of the *Dictyostelium discoideum* wild-type strain AX2-214, or mutant cells derived from it, were cultivated at 21°C in nutrient HL5 medium (Formedium), either in shaken culture at 150 rpm or in Petri dishes without shaking. To induce starvation, cells were washed twice in 17 mM Soerensen’s phosphate buffer (PB), pH 6.0, and shaken at a density of 10^7^ cells per ml in the buffer.

To generate *ndrC*-null mutants, wild-type AX2 cells were transformed with the gene replacement construct (Figure 2A) by electroporation using a Bio-Rad gene pulser at 0.8-0.9 kV and 3 μF, and 4-mm cuvettes. Independent transformants were selected by addition of 7.5 μg/ml of Blasticidin-S (ICN Biomedicals Inc.). Transformants were cloned by spreader dilutions on lawns of non-pathogenic *Klebsiella aerogenes*. Three independent NdrC-knockout clones were identified by testing genomic DNA for insertion of the resistance cassette into the *ndrC* gene by PCR.

For expression of fluorescently tagged Ras GTPases, AX2 wild-type and NdrC-null cells were transformed by electroporation with pDEX derived plasmids enabling the expression of GFP-RasB, GFP-RasB-G12T, GFP-RasG and GFP-RasG-G12T. Transformants were selected by addition of 10 μg/ml of G418 (Sigma-Aldrich) or 7.5 μg/ml Blasticidin-S (ICN Biomedicals Inc.), and cloned.

### Live-cell microscopy

Cells were transferred into an open chamber and washed twice with Soerensen phosphate buffer. Confocal images were taken using an inverted LSM 510 Meta confocal microscope (Zeiss) equipped with a 63× Neofluar 1.4 or a 100× Neofluar 1.3 oil immersion objective. For excitation, the 488-nm argon ion laser line and the 543-nm as well as the 633-nm helium neon laser lines were used, and emission was collected using 505–530 nm, 585–615 nm band-pass or a 650 nm long-pass filter.

### Immunofluorescence

For immunolabeling, AX2 wild-type or *ndrC*-null cells, settled onto glass coverslips, were fixed with a mixture of 15% (v/v) of saturated picric acid and 2% (w/v) paraformaldehyde in 10 mM Pipes-HCl, pH 6.0, at room temperature for 20 min, and post-fixed with 70% ethanol for 10 min. α-tubulin was detected using monoclonal rat antibodies (YL1/2) [43] and Alexa Fluor 568-conjugated goat anti-rat IgG (Invitrogen). For visualization of filamentous actin, cells were stained with Alexa Fluor 488-phalloidin (Molecular Probes). DNA was visualized either with TO-PRO-3 or with DAPI.

Polyclonal antibodies were generated against GST-tagged NdrC-RBD. The sequence encoding amino acid residues 1 to 299 of NdrC was cloned into the bacterial expression vector pGEX-6P1 (GE Healthcare). Purification of the GST-fusion protein was performed using standard procedures, and the recombinant protein was used to immunize a female white New Zealand rabbit together with the adjuvant Gerbu100 (Gerbu Biochemicals).

### GST-RBD pull-down assay of endogenous Ras proteins

The binding of endogenous activated Ras proteins to the GST-Ras Binding Domain (GST-RBD) of *Dictyostelium* NdrC was determined as described previously [39]. 350 μl of *Dictyostelium* wild-type AX2 or *ndrC*-null cell suspension (5 × 10^7^ cells/ml) were lysed by mixing with an equal volume of 2× lysis buffer. 400 μg of protein lysate were incubated with 100 μg of GST-RBD and glutathione sepharose beads (GE Healthcare) at 4°C for 1 hour. 50 μl of 1× SDS gel loading buffer was added to the pelleted beads, and the suspension was boiled for 5 min. Samples were subjected to SDS-PAGE, and Western blots were probed with polyclonal antibodies directed against RasB, RasG, RasC, or Rap1. Equal sample loading was verified by staining of a duplicate gel with Coomassie Blue.

### Interaction of His-tagged Ras and GST-NdrC

To produce 6xHis-tagged Ras-GTPases, wild-type RasG, RasC , RasB, RasS, Rap1 and RasD, and the constitutively activated forms of RasG (G12T), RasC (G13T), RasB (G15T), RasS (G12T), Rap1 (Q65E) and RasD (G12T) were cloned into pET-21a (Novagen) [41]. To express GST-NdrC, a fragment encoding amino acid residues 1–299 of NdrC was cloned into pGEX6P-1. Recombinant proteins were expressed in *Escherichia coli* BL21 (DE3) codonplus-RIL (Stratagene), and protein concentrations were determined using the DC Protein Assay (Bio-Rad). His-Ras/RBD-NdrC-GST interaction was examined as described previously [41]. In brief, 400 μg of purified His-tagged Ras protein were incubated with 100 μg of GST-RBD on glutathione sepharose beads and tumbled in binding buffer at 4°C overnight. Beads were washed three times and analyzed by SDS-PAGE, and Western blots were probed with anti-His tag monoclonal antibody (Santa Cruz Biotechnology).

## Abbreviations

aa: Amino acids; GST: Glutathione-S-transferase.

## Competing interests

The authors declare that they have no competing interests.

## Authors’ contributions

AMT and GW designed and supervised research; AMT, PMK and PB performed research; MS advised experiments and contributed financially; AMT, PMK, PB and GW analyzed data; AMT and GW wrote the manuscript. All authors read and approved the final manuscript.

## Supplementary Material

Additional file 1: Figure S1 Comparison of NdrC to other LATS/NDR kinases and LATS/NDR kinase sequence signatures of *Dictyostelium* NdrC. **A**. Sequence identities of the catalytic domain of *D. discoideum* NdrC with the catalytic domains of *D. discoideum* NdrD, NdrA and NdrB, in comparison to *Homo sapiens* NDR1 and 2, and LATS1 and 2, and the *Drosophila melanogaster* LATS-homolog Warts. Numbers indicate per cent identity within the catalytic domains compared to NdrC catalytic domain determined by BLASTp. **B**. Sequence signatures of *Dictyostelium* NdrC. The NTR region (amino acid residues 650 to 710) carries a conserved phosphorylation site at threonine 703. The catalytic domain (subdomains I-X; amino acid residues 718 to 1019) contains an AGC-kinase specific insert (I; amino acid residues 867 to 913) as well as an adjacent activation segment (AS; amino acid residues 914 to 928) containing a conserved regulatory phosphorylation site at serine 917. The conserved hydrophobic motif (HM; amino acid residues 1091 to 1099) corresponds to the consensus sequence F_X_X_Y/F_T_Y/F_K/R carrying a putative phosphorylation site at threonine 1095 [1]. Click here for file

Additional file 2: Figure S2 Growth rates of ndrC-null cells compared to wild-type. **A**. Growth rates of *ndrC*-null cells compared to wild-type cells in rich medium under shaking conditions. **B**. Growth of wild-type and *ndrC*-null cells on bacterial lawns of non-pathogenic *K. aerogenes* on agar plates. Click here for file

Additional file 3: Figure S3 NdrC co-purifies with centrosomes. Centrosomes were isolated from cells expressing GFP-NdrC by purification of nuclei followed by pyrophosphate treatment and sucrose density centrifugation. The nuclei fraction with the associated centrosomes was disintegrated by pyrophosphate and passage through a 5-μm mesh polycarbonate filter. Centrosomes were isolated via two consecutive sucrose step gradients of 80% and 50%, followed by 80%, 70%, 55% and 50% steps in SW-40 tubes (Beckman) centrifuged at 55,000 × g for 1 h at 4°C. Immunostaining of methanol-fixed centrosomes was performed with monoclonal anti-CP224 antibodies [31]. The primary antibodies were visualized with Alexa Fluor-568 anti-mouse IgG (Invitrogen). Centrosomes labeled by anti-CP224 antibodies are red, those containing GFP-NdrC are green, and those containing both labels are yellow. Very similar results were obtained with centrosomes isolated from wild-type cells and immunostaining with polyclonal anti-NdrC-RBD antibodies and Alexa Fluor-488 anti-rabbit IgG. Click here for file

Additional file 4: Figure S4 Localization of GFP-NdrC(435–1312). **A**. Scheme of the GFP-tagged NdrC (435-1312) construct. **B**. Live-cell imaging of a *Dictyostelium* wild-type cell expressing GFP-NdrC(435–1312). Bar, 5 μm. Click here for file

Additional file 5: Figure S5 Immunolocalization of RasG in wild-type cells. Wild-type cells were fixed and immunostained with polyclonal antibodies directed specifically against RasG. Primary antibodies were detected with Alexa Fluor-488 anti-rabbit IgG (green). Nuclei were visualized by staining with DAPI (blue). Bar, 5 μm. Click here for file
