# Supplementary figures and images for: Regulation of a LATS-homolog by Ras GTPases is important for the control of cell division
Source: BMC Cell Biol. 2014 Jul 1;15:25. doi: 10.1186/1471-2121-15-25 (PMC4120859; doi:10.1186/1471-2121-15-25)

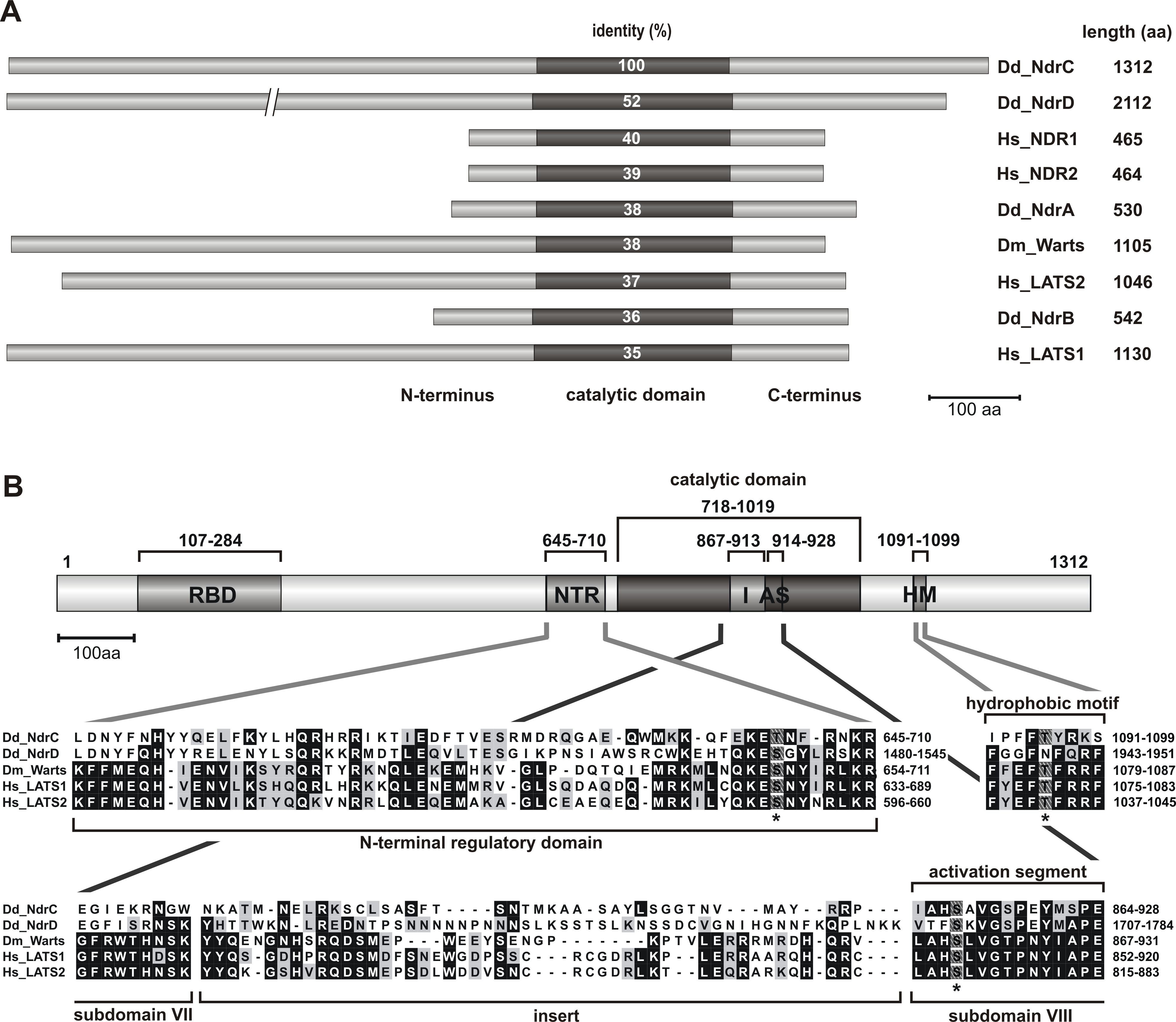

Supplement: Additional file 1: Figure S1 — Comparison of NdrC to other LATS/NDR kinases and LATS/NDR kinase sequence signatures of Dictyostelium NdrC. A. Sequence identities of the catalytic domain of D. discoideum NdrC with the catalytic domains of D. discoideum NdrD, NdrA and NdrB, in comparison to Homo sapiens NDR1 and 2, and LATS1 and 2, and the Drosophila melanogaster LATS-homolog Warts. Numbers indicate per cent identity within the catalytic domains compared to NdrC catalytic domain determined by BLASTp. B. Sequence signatures of Dictyostelium NdrC. The NTR region (amino acid residues 650 to 710) carries a conserved phosphorylation site at threonine 703. The catalytic domain (subdomains I-X; amino acid residues 718 to 1019) contains an AGC-kinase specific insert (I; amino acid residues 867 to 913) as well as an adjacent activation segment (AS; amino acid residues 914 to 928) containing a conserved regulatory phosphorylation site at serine 917. The conserved hydrophobic motif (HM; amino acid residues 1091 to 1099) corresponds to the consensus sequence F_X_X_Y/F_T_Y/F_K/R carrying a putative phosphorylation site at threonine 1095 [1]. [file 1471-2121-15-25-S1.jpeg]

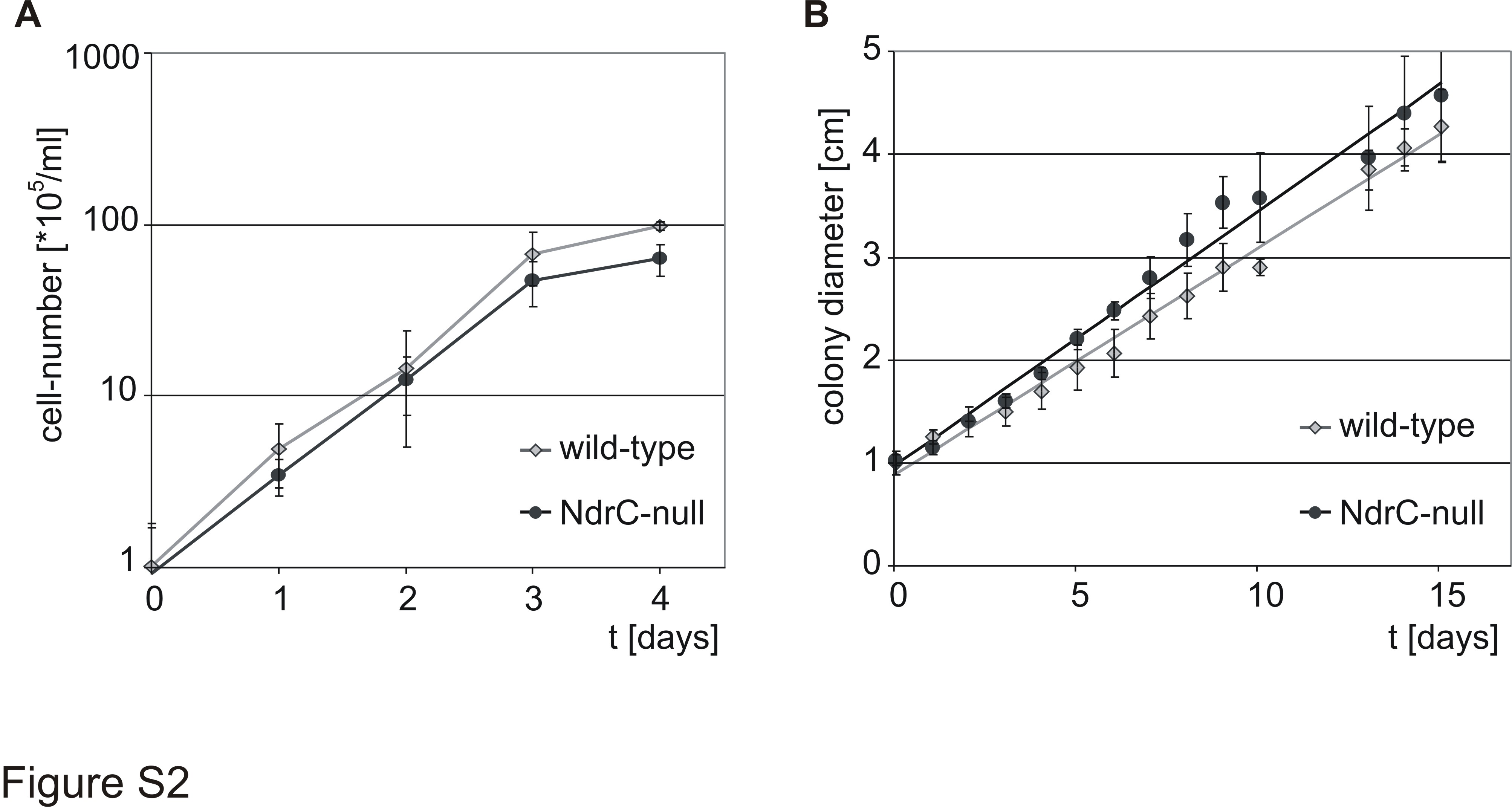

Supplement: Additional file 2: Figure S2 — Growth rates of ndrC-null cells compared to wild-type. A. Growth rates of ndrC-null cells compared to wild-type cells in rich medium under shaking conditions. B. Growth of wild-type and ndrC-null cells on bacterial lawns of non-pathogenic K. aerogenes on agar plates. [file 1471-2121-15-25-S2.jpeg]

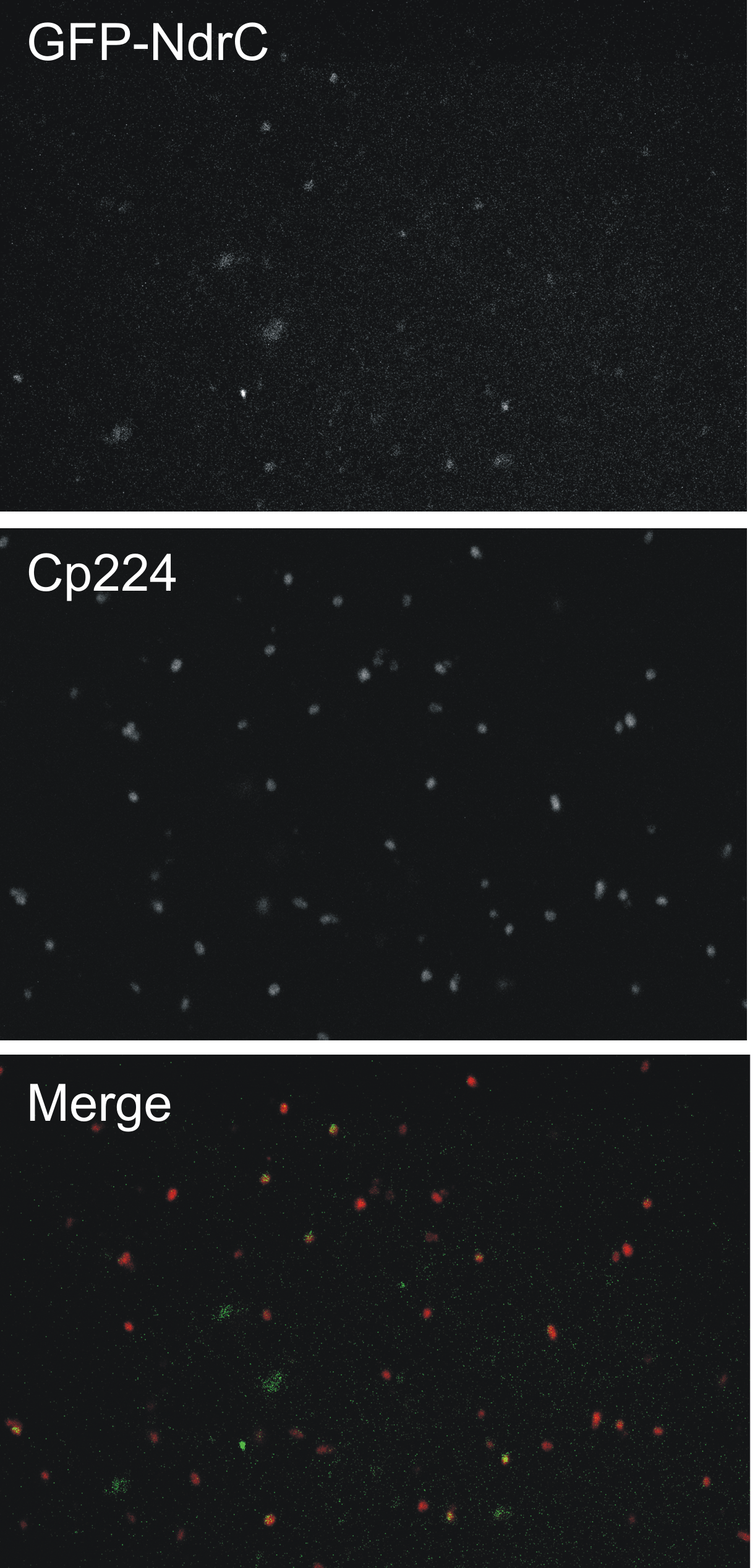

Supplement: Additional file 3: Figure S3 — NdrC co-purifies with centrosomes. Centrosomes were isolated from cells expressing GFP-NdrC by purification of nuclei followed by pyrophosphate treatment and sucrose density centrifugation. The nuclei fraction with the associated centrosomes was disintegrated by pyrophosphate and passage through a 5-μm mesh polycarbonate filter. Centrosomes were isolated via two consecutive sucrose step gradients of 80% and 50%, followed by 80%, 70%, 55% and 50% steps in SW-40 tubes (Beckman) centrifuged at 55,000 × g for 1 h at 4°C. Immunostaining of methanol-fixed centrosomes was performed with monoclonal anti-CP224 antibodies [31]. The primary antibodies were visualized with Alexa Fluor-568 anti-mouse IgG (Invitrogen). Centrosomes labeled by anti-CP224 antibodies are red, those containing GFP-NdrC are green, and those containing both labels are yellow. Very similar results were obtained with centrosomes isolated from wild-type cells and immunostaining with polyclonal anti-NdrC-RBD antibodies and Alexa Fluor-488 anti-rabbit IgG. [file 1471-2121-15-25-S3.tiff]

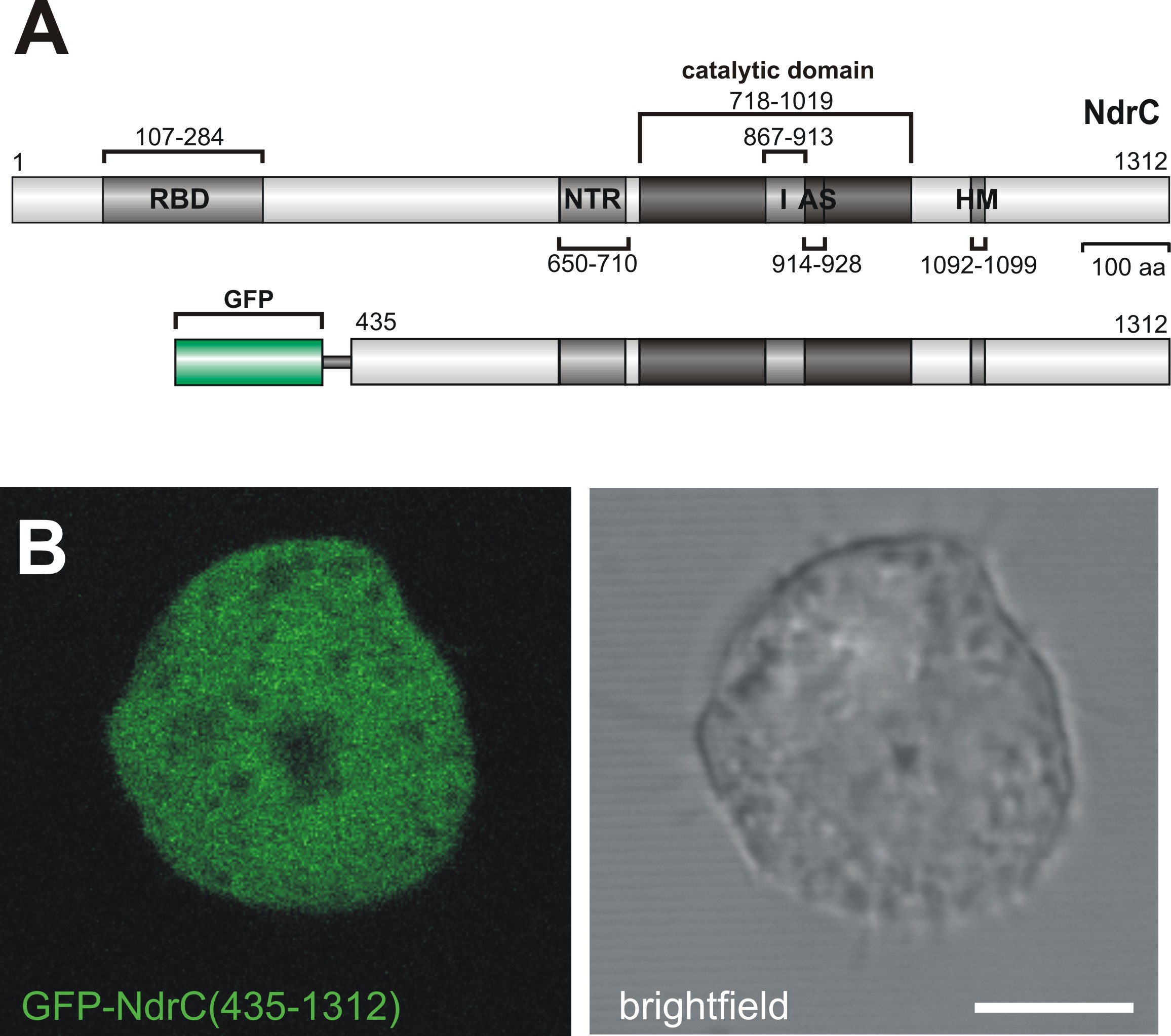

Supplement: Additional file 4: Figure S4 — Localization of GFP-NdrC(435–1312). A. Scheme of the GFP-tagged NdrC (435-1312) construct. B. Live-cell imaging of a Dictyostelium wild-type cell expressing GFP-NdrC(435–1312). Bar, 5 μm. [file 1471-2121-15-25-S4.jpeg]

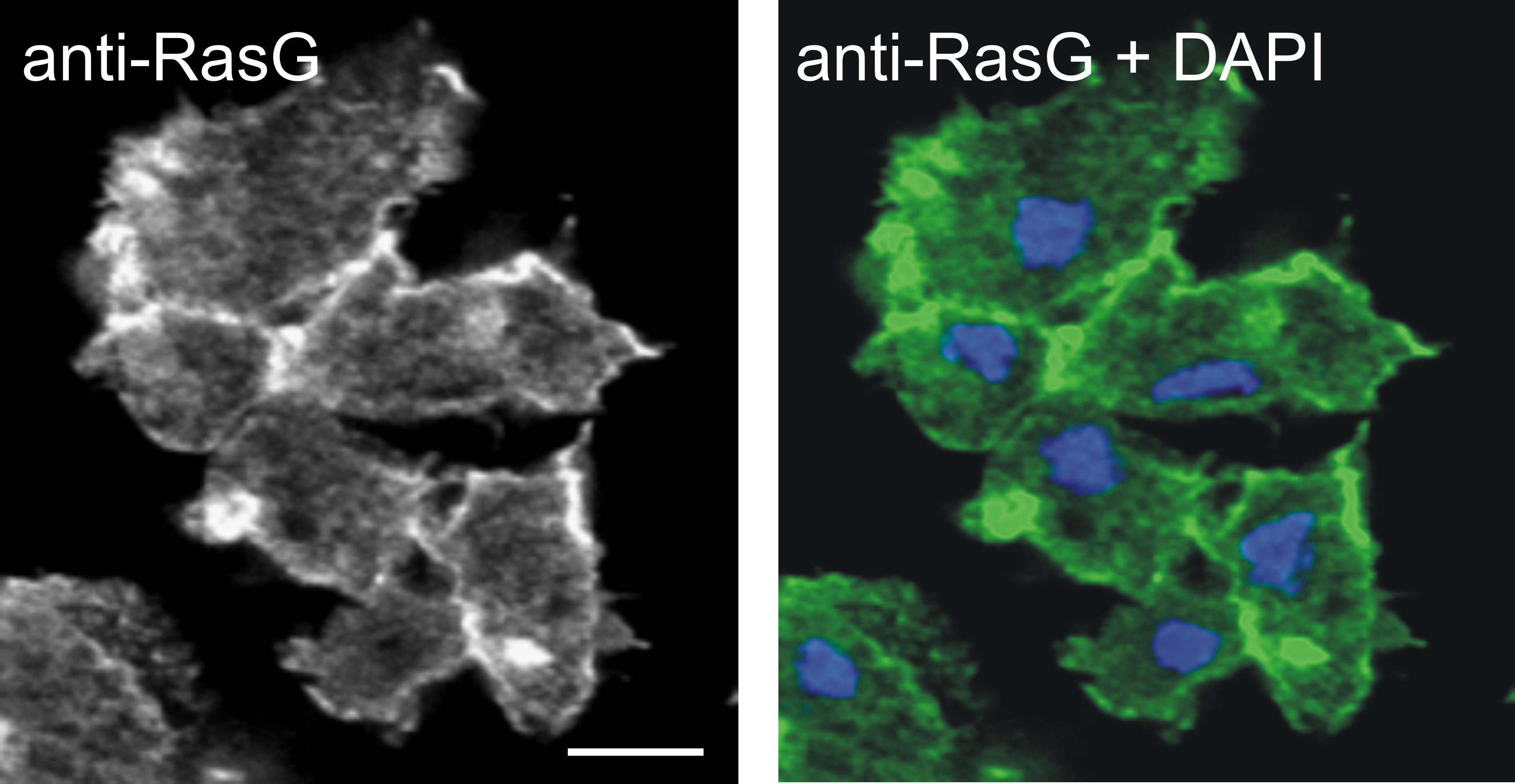

Supplement: Additional file 5: Figure S5 — Immunolocalization of RasG in wild-type cells. Wild-type cells were fixed and immunostained with polyclonal antibodies directed specifically against RasG. Primary antibodies were detected with Alexa Fluor-488 anti-rabbit IgG (green). Nuclei were visualized by staining with DAPI (blue). Bar, 5 μm. [file 1471-2121-15-25-S5.tiff]
